# Employing the CRISPR-Cas System for Clonal Hematopoiesis Research

**Published:** 2020-11-30

**Authors:** Hayato Ogawa, Soichi Sano, Kenneth Walsh

**Affiliations:** 1 Department of Cardiovascular Research, University of Virginia, Charlottesville, Virginia, United States; 2 Department of Hematovascular Biology, University of Virginia, Charlottesville, Virginia, United States; 3 Department of Cardiology, Graduate School of Medicine, Osaka City University, Osaka, Japan

**Keywords:** Clonal hematopoiesis, CRISPR-Cas system, Genome editing, Cell type specificity, DNA damage response, Somatic mosaicism, Hematology

## Abstract

Clonal hematopoiesis is a state in which substantial fraction of hematopoietic stem cells acquire mutations in specific driver genes and expand in the absence of an overt hematological malignancy. Recent clinical studies have shown that clonal hematopoiesis increases likelihood of hematological malignancy and cardiovascular disease. While clinical studies have identified countless candidate driver genes associated with clonal hematopoiesis, experimental studies are required to evaluate causal and mechanistic relationships with disease processes. This task is technically difficult and expensive to achieve with traditional genetically engineered mice. The versatility and programmability of CRISPR-Cas system enables investigators to evaluate the pathogenesis of each mutation in experimental systems. Technical refinements have enabled gene editing in a cell type specific manner and at a single base pair resolution. Here, we summarize strategies to apply CRISPR-Cas system to experimental studies of clonal hematopoiesis and concerns that should be addressed.

## CRISPR-CAS GENOME EDITING TO STUDY CLONAL HEMATOPOIESIS

Somatic mutations accumulate over time and are an inevitable consequence of aging. Actively dividing cells, such as hematopoietic stem cells, are likely to acquire mutations at a high frequency [1]. While most mutations are neutral or deleterious, some mutations will confer a fitness advantage to the cell. Clonal hematopoiesis, or clonal hematopoiesis of indeterminate potential (CHIP), is a state in which mutant hematopoietic stem cells undergo a clonal expansion in the absence of an overt hematological abnormality [2]. While this condition increases the likelihood of a hematological malignancy, clonal hematopoiesis has been observed to also have adverse effects on extra-hematological tissues, and this has attracted attention of researchers who are typically not engaged in the field of hematology. A notable example is in the field of cardiology, where growing evidence shows that clonal hematopoiesis increases the incidence of coronary artery disease and stroke, and worsens prognosis in the heart failure patients [3–5].

To investigate the mechanisms by which clonal hematopoiesis contributes to pathogenicity, genetically engineered mouse models have been employed [5–8]. However, these approaches can be limited by the availability of appropriated transgenic mouse lines. This limitation is amplified by epidemiological data showing that clonal hematopoiesis can result from a number of putative “driver genes” (∼60) that are recurrently mutated in hematologic malignancies [4,9]. Furthermore, whole genome sequencing of 11,262 individuals without hematological malignancy reveal that >80% of clonal hematopoiesis driver mutations may exist outside of the genes recurrently mutated in hematological malignancy, and these candidate “drivers” of clonal expansions have not been identified or characterized thus far [10]. Therefore, alternative strategies to investigate new candidate driver mutations at a genome-wide scale is required to more rapidly develop a better understanding of the pathophysiology of clonal hematopoiesis. In this short commentary, we discuss the recent applications of CRISPR-Cas systems in clonal hematopoiesis research.

The versatility and programmability of clustered regularly interspaced short palindromic repeats (CRISPR)-based technologies have been applied in biological and biotechnological research. Since its first application to edit the mammalian genome in 2013, the application of this technology has grown exponentially, leading to the awarding of Nobel Prize for the discovery of this genetic tool in 2020. CRISPR-Cas systems involve two key components: Cas endonucleases and engineered single guide RNAs (sgRNA) that direct Cas nuclease to the target site on the genome. The sgRNAs have complementary sequence to the target regions within the genome, which enables the sequence-specific recognition of DNA. In a simple system, Cas nuclease introduces a double strand DNA break at the target site. These breaks are repaired by non-homologous end joining (NHEJ) and results in the random introduction of insertions and deletions. Alternatively, double strand breaks can be repaired more precisely through homology-directed repair (HDR) in the presence of donor DNA [11].

CRISPR-Cas methodology has allowed advances in our understanding of hematology and oncology. The Ebert lab applied CRISPR technology to introduce mutations in mouse primary hematopoietic stem/progenitor cells (HSPC) to understand the processes that contribute to hematologic malignancies [12]. In this study, they constructed lentivirus vectors to deliver both the sgRNA and a gene encoding the Cas9 protein in a single vector. Although cumbersome, this approach led to the successful introduction of clinically relevant mutations in a variety of genes, including DNA methyltransferase 3 alpha (*Dnmt3a*) and tet methylcytosine dioxygenase 2 (*Tet2*), within HSPC, and their introduction to mice to establish blood cancer models. Our lab repurposed this method to the study impact of clonal hematopoiesis on cardiovascular disease, demonstrating that *Tet2* or *Dnmt3a* loss-of-function mutations in hematopoietic cells can accelerates angiotensin II-induced heart failure [13]. To improve the efficiency of this procedure, we showed that it is possible to decouple the two major components of CRISPR-Cas systems: i.e. the effector Cas nucleases (mainly spCas9) and the sgRNA. Specifically, it is possible to employ HSPC isolated from Cas9-transgenic mice, and thereby diminish the size of the lentivirus vector. Thus, the lentivirus only needs to deliver the sgRNA to enable CRISPR-based genome editing, and this is more efficient and less laborious [14,15].

## ADDRESSING NEW GENE EDITING CHALLENGES IN CLONAL HEMATOPOIESIS RESEARCH

A current challenge is a need to recreate somatic mutation seen in individuals with clonal hematopoiesis at a single-base resolution. Some clonal hematopoiesis driver genes contain hotspot sites where particular mutations are enriched. An example is missense mutations at R882 residue in *DNMT3A* gene that most commonly results in an arginine to histidine change (i.e. *DNMT3A*^*R882H*^).

This is important because it has been suggested that mutations at different sites within the same driver gene can differentially impact the functions of the mutant proteins [16,17]. These concerns highlight the needs to exactly recapitulate the human mutations in the mouse models. Currently, the widely used systems rely mainly on the stochastic introduction of mutations due to NHEJ. This is far from ideal as it generates a complicated heterogeneous mixture of mutant clones in every experiment. However, newly developed technology such as “base editing” or “prime editing” can be used to introduce specific nucleotide alterations in the genome [18,19]. In base editing, a DNA deaminase such as cytidine deaminase, is fused with catalytically inactivated Cas9 (dCas) and is directed by a sgRNA to convert a single base pair at the target site. This results in the generation of a targeted point mutation and avoids DNA double strand breaks. In prime editing, a fusion of Cas9 nickase (a partially inactivated Cas9, which introduces a single-strand cut instead of double strand breaks) is combined with reverse transcriptase. Gene editing is directed by a prime-editing gRNA (pe gRNA) that includes a template sequence for precise genome manipulation. After introduction of a nick upstream of the target sequence, the reverse transcriptase extends the cleaved strand based on the template sequence in the pe gRNA, and DNA is repaired using the extended nucleotides as a template.

**Figure 1:**
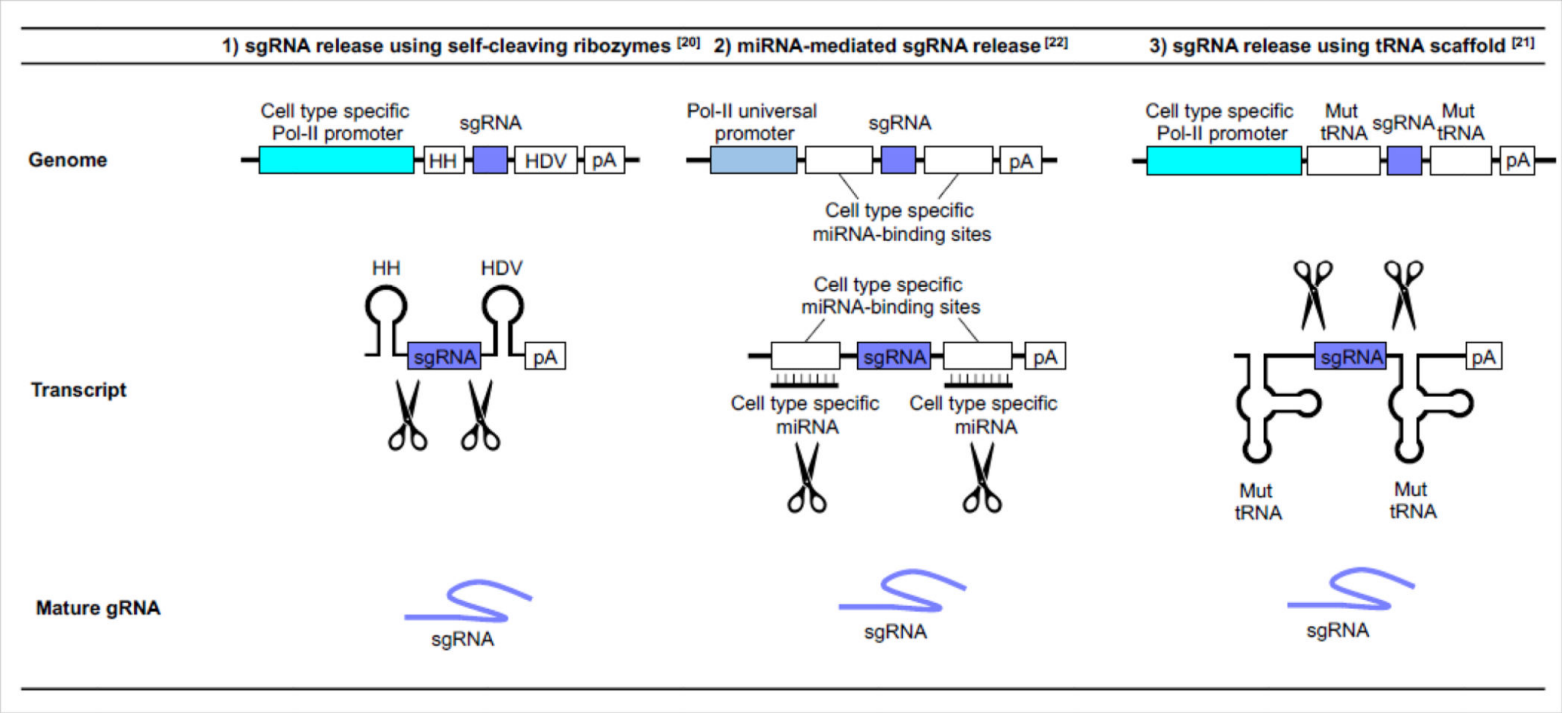
Strategies for cell type specific sgRNA expression. Cell type specific sgRNA expression can be achieved by 1) the combination of a cell type specific RNA polymerase-II (Pol-II) promoter and sgRNA flanked with self-cleaving ribozymes: e.g. hammerhead (HH) ribozyme and hepatitis delta virus (HDV) ribozyme, 2) transcript processing by cell type specific miRNA or 3) the combination of cell type specific Pol-II promoter and sgRNA flanked with a mutant transfer RNA (Mut tRNA) which has minimal promoter activity and can maximize processing activity. **Abbreviation:** pA: Polyadenylation Signal.

Another challenge is the introduction of mutations in a cell type-specific manner. The somatic mutations associated with clonal hematopoiesis occur in HSPC, and they are then transmitted to the different blood cell progeny. However, blood cell populations are diverse, and the effects of somatic mutations can be divergent depending upon the cell type. Therefore, cell-type specific approaches are required to elucidate mechanistic details of the pathology that results from clonal hematopoiesis. There are several approaches to achieve cell-type specific gene perturbation by CRISPR-Cas systems. The expression of sgRNA is most often under the control of powerful RNA polymerase-III promoters such as U6 or H1 promoters, which are constitutively active. Alternatively, sgRNA expression can be directed by cell type-specific RNA polymerase-II (Pol-II) promoters. As sgRNAs themselves are not transcribed by Pol-II, the strategy is to transcribe RNA precursors that contain sgRNA sequence, which are then processed to release mature sgRNAs (Figure 1) [20,21]. While progress has been made, the activity of sgRNA produced from Pol-II promoters tends to be suboptimal. This is possibly due to inefficient sgRNA processing from their precursors, and the diminished transcriptional activity of the cell type-restricted promoters. Thus, further testing and optimization are required. It might be possible to improve efficiency by enhancing sgRNA stability. Alternatively, it should also be possible to achieve cell type-specific sgRNA expression by taking advantage of the tissue-specific machinery that can process sgRNA precursors. An example of this approach includes a method that repurpose the cell-type specific expression of miRNAs. In a particular case, the sgRNA sequence is flanked by the sequences that are targeted by cell-type specific miRNAs (Figure 1) [22]. It should be possible to apply these techniques to study hematopoietic lineages using the appropriate lineage-specific miRNA targets.

## CRISPR-CAS CONCERNS THAT NEED TO BE ADDRESSED

There are numerous potential pitfalls associated with the application of the CRISPR-Cas system in disease models. In addition to the widely recognized possibility of off-target cleavage activity of CRISPR-Cas [23], investigators have come to better appreciate the consequences of on-target DNA breaks. Genomic cleavage by Cas9 nuclease has been reported to activate p53-mediated DNA damage response (DDR), which causes growth disadvantage/arrest [24]. More recently, it has been reported that the expression of Cas9 protein itself elicits the DDR independently of DNA cleavage [25]. These responses appear to be specific to cell type. Thus, an evaluation of whether these processes are operational in hematopoietic cells is required through the development of well-designed experiments. In particular, these issues could be important in future investigations of therapy-related clonal hematopoiesis that occurs in cancer survivors who have undergone therapy with genotoxic agents, which frequently results from HSPCs harboring mutations in TP53 and PPM1D that encode components of the DDR pathway [26,27]. Subtle differences in the DDR caused by CRISPR-mediated NHEJ can be amplified under disease conditions and could confound the interpretation of experimental results. Thus, appropriate controls should be employed when considering the CRISPR-Cas system for clonal hematopoiesis research. For example, cells from Cas9-negative strains can be employed as controls to assess the potential adverse effects of ectopic Cas9 expression on the experimental system under investigation. To assess the possibility of DDR activation by DNA cleavage of the target gene per se, controls could employ cells that are edited by an sgRNA that targets the ROSA26 locus or another non-essential region of the genome. Ultimately, it would be ideal to corroborate key findings by employing traditional transgenic approaches for gene manipulation.

Finally, CRISPR-Cas systems are typically employed to generate small indels in the genome to ablate specific genes of interest. However, due to error-prone NHEJ, these repair processes can sometimes result in large deletions and complex chromosomal rearrangement [28] as well as entire chromosome loss [29]. Thus, as mentioned in previous section, more precise genome editing approaches, perhaps at a single-base resolution as described in previous section, could avoid these potential pitfalls.

## CONCLUSION

It is becoming increasingly apparent that clonal hematopoiesis is prevalent in the elderly population and associated with mortality and age-associated diseases. This condition can be caused by a multitude of mutations in candidate driver genes within HSPC. CRISPR-Cas technology enables investigators to evaluate the pathological significance of countless candidate mutation, which would nearly be impossible using traditional transgenic mouse approaches. However, there are challenges and pitfalls in the application of this technology that investigators should consider, and experiments need to employ appropriate controls. It is expected that these issues will be resolved given the advances in this methodology and the interest in the scientific community.
